# Supplementary material for: Evolution of satellite plasmids can prolong the maintenance of newly acquired accessory genes in bacteria
Source: Nat Commun. 2019 Dec 20;10:5809. doi: 10.1038/s41467-019-13709-x (PMC6925257; doi:10.1038/s41467-019-13709-x)
Supplement: Supplementary file 1 — Supplementary Information [file 41467_2019_13709_MOESM1_ESM.pdf]

Supplementary Information for

Evolution of satellite plasmids can prolong the maintenance of newly acquired accessory genes in bacteria

Xue Zhang, Daniel E. Deatherage, Hao Zheng, Stratton J. Georgoulis, Jeffrey E. Barrick

Jeffrey E. Barrick
Email: jbarrick@cm.utexas.edu

This PDF file includes:

Supplementary Figures 1 to 5
Supplementary Table 1

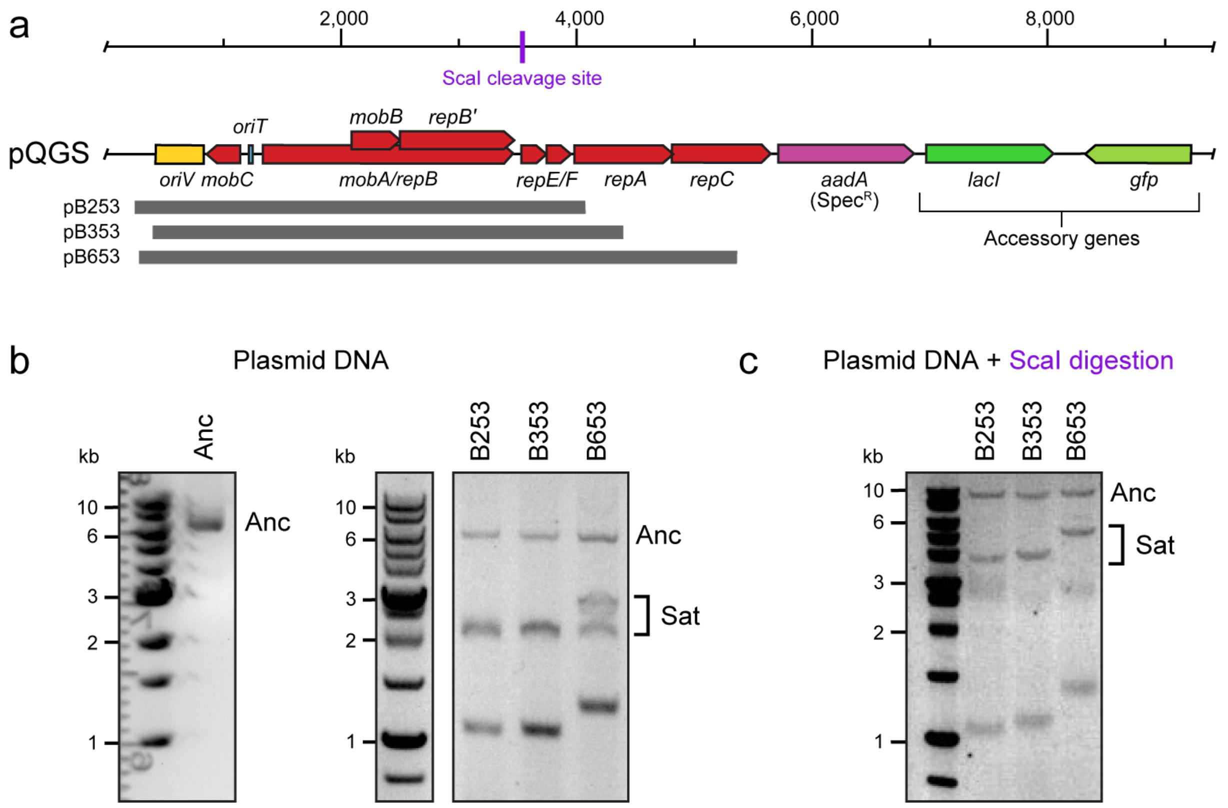

Supplementary Figure 1. Direct visualization of satellite plasmids in evolved *E. coli* isolates**. a** Map of pQGS plasmid showing the single site cleaved by the restriction enzyme ScaI and the regions that are preserved in the satellite plasmids that are present in evolved strains B253, B353, and B653. **b** Total plasmid DNA isolated from the ancestor strain (Anc) and each of these evolved strains separated by gel electrophoresis. **c** Total plasmid DNA isolated from each of these evolved strains, digested with ScaI, and then separated by gel electrophoresis. After the circular pQGS ancestor plasmid present in these samples (Anc) is linearized by ScaI, the mobility of its band decreases, and it runs at the expected size of 9.3 kb relative to the DNA ladder. Similarly, the mobility of the satellite plasmid bands (Sat) decreases upon cleavage, which is consistent with the satellite plasmids being maintained as circular DNA molecules in these cells. After linearization, the satellite plasmid bands run at sizes that agree with the results of sequencing PCR products to determine which regions of the ancestral plasmid were deleted in each one. The nature of the molecules observed in the 1.0-1.5 kb size range relative to the DNA ladder is unclear. They are unaffected by ScaI treatment, and their apparent sizes track with the relative sizes of the satellite plasmids in each strain. They could be wholly or partially single-stranded DNA intermediates related to plasmid replication and/or transfer. Source data are provided as a Source Data file.

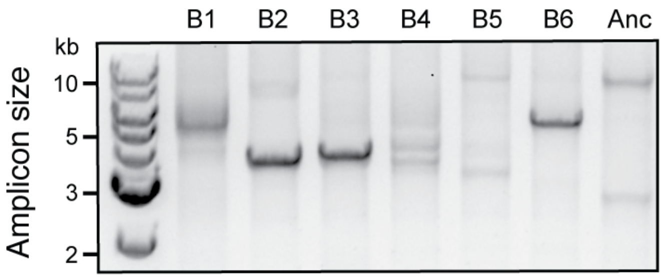

Supplementary Figure 2. Satellite plasmids evolved in all *E. coli* populations. Plasmid DNA was isolated from all six populations (B1–B6) of the *E. coli* evolution experiment on day 5. Then the PCR assay described in Figure 1 was used to generate linearized amplicons to detect fragments with reduced sizes indicating that satellite plasmids were present in these genetically heterogenous populations. Source data are provided as a Source Data file.

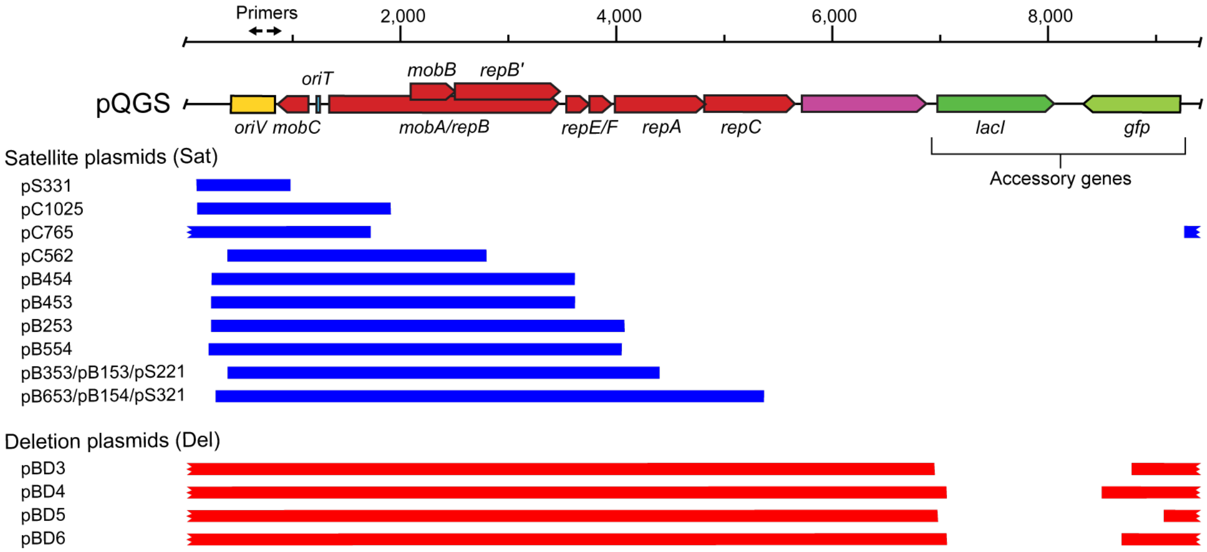

Supplementary Figure 3. Maps of evolved plasmids. Bars show regions preserved in plasmids that evolved in different *E. coli* (pB) and *S. alvi* (pS) populations cultured in the laboratory or in *S. alvi* populations that colonized the guts of honey bees reared in different laboratory enclosures (pC). Satellite plasmids are displayed in blue. Deletion plasmids are displayed in red. The plasmid naming scheme is described in the legend to Supplementary Table 1.

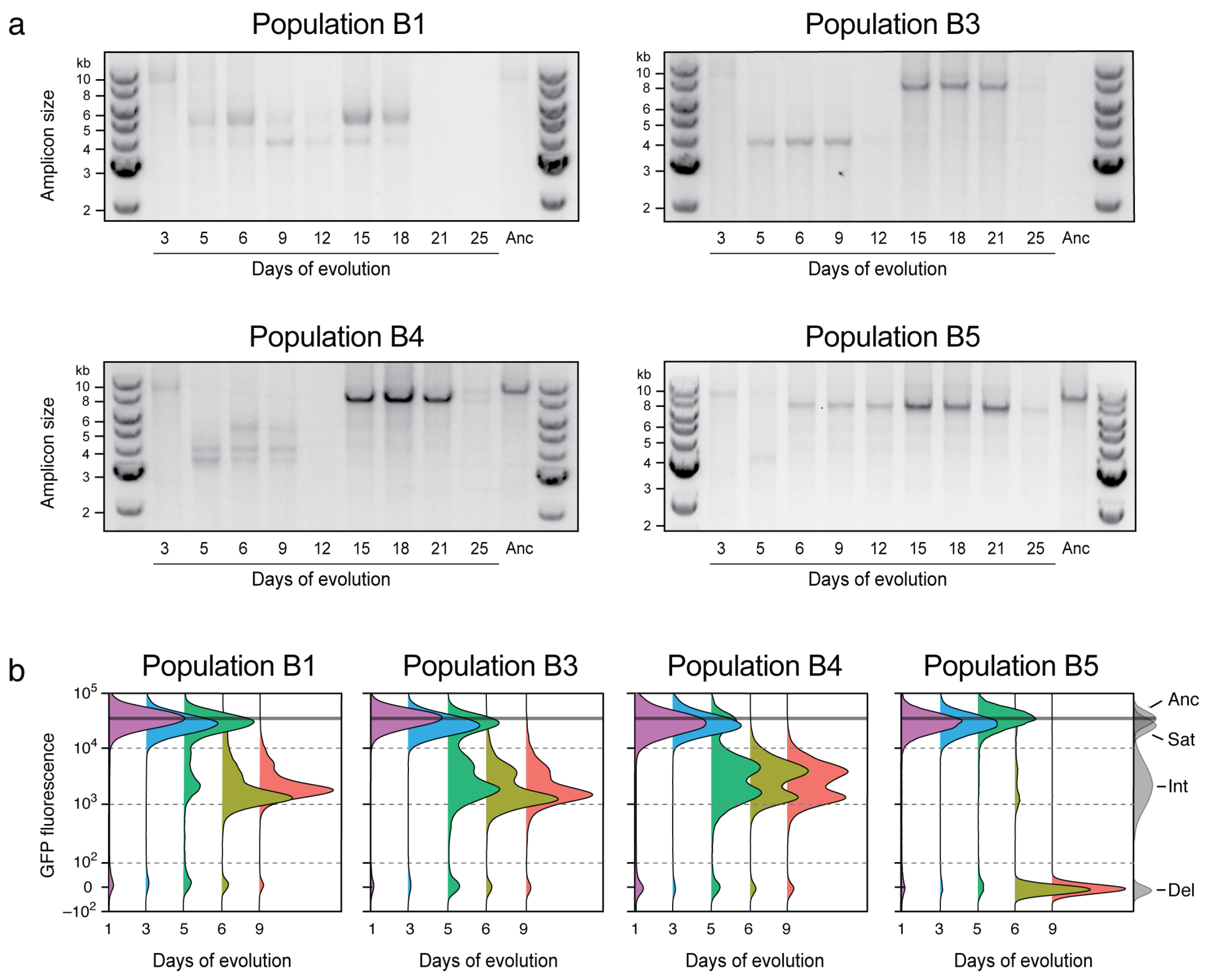

Supplementary Figure 4. Time courses of plasmid evolution in the remaining *E. coli* populations**.** **a** Appearance and persistence in populations B1, B3, B4, and B5 of satellite plasmids and plasmids with accessory-gene deletions that also reduce the size of the linearized plasmid PCR amplicon. **b** Distributions of GFP expression in cells in each population determined using flow cytometry. Source data are provided as a Source Data file.

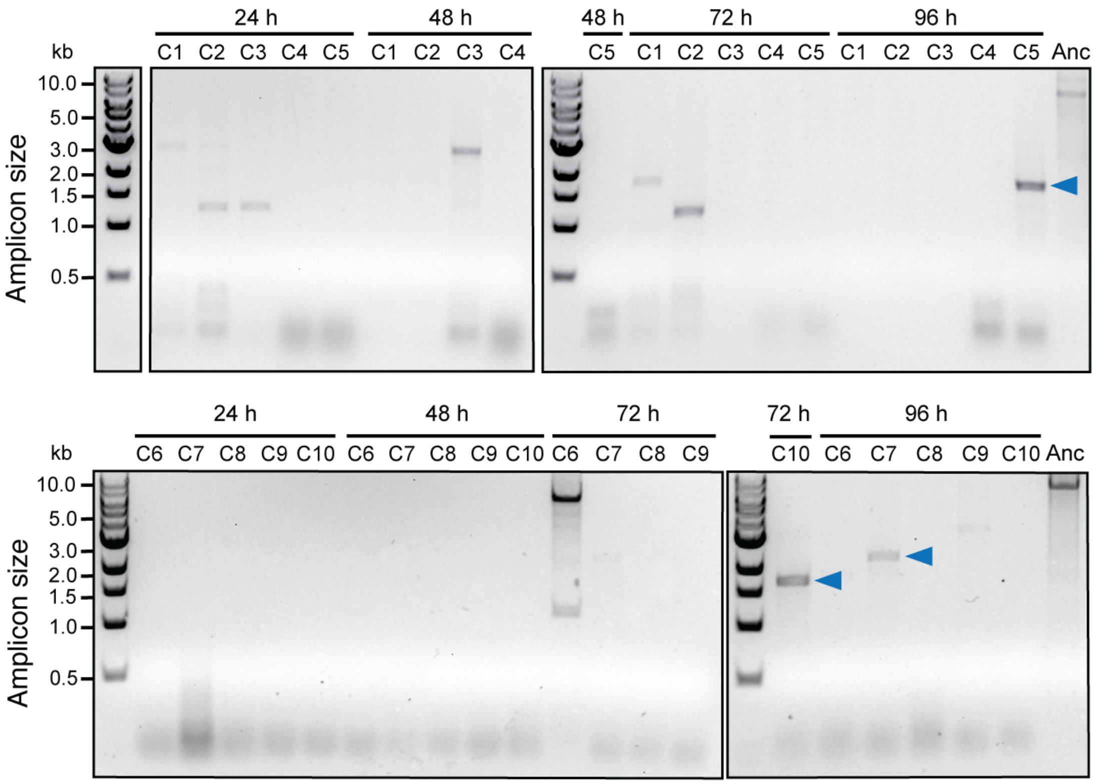

Supplementary Figure 5. Evolution of satellite plasmids in *S. alvi* populations colonizing honey bee guts. Multiple bees were reared in a total of ten separate enclosures in two sets of experiments conducted at different times. One bee from each enclosure (C1–C5 or C6–C10) was sacrificed at each of the indicated time points. DNA was isolated from each bee gut and subjected to the PCR assay to detect linearized amplicons from plasmids with deletions. Blue arrows in all panels are bands that were Sanger sequenced to validate that they represent satellite plasmids (Supplementary Fig. 2 and Supplementary Table 1). Source data are provided as a Source Data file.

Supplementary Table 1. Sequence context of pQGS deletions

Evolved plasmid	Plasmid size (bp)	Endpoint coordinates	Flanking sequences (microhomology)
**Satellite plasmids (Sat)**			
pS331	854	964	GATCCTCCgGCCaCt | CGCTGCTGTTCACCT
		111	CATGCTCCaGCCgCc | CGCATTGGAGAAATT
pC1025	1793	1900	GCGGGCTGGCCACGA | CGCCCGCATTGACCA
		108	AAGCCATGCTCCAGC | CGCCCGCATTGGAGA
pC765	1863	1708	CCCGCACTGCCACcT | GATGATCTCCGAGCG
		9243	GCGACGAATTGCCAC | gTTGTCGCAGTGTCT
pC562	2396	2783	GGCCGGGAGCCTGCC | CTGGTAGTGGAAACC
		388	GCCGAAATGCCTGCC | GTTGCTAGACATTGC
pB454	3359	3606	GGTTGCCCGGTGGCT | tTGGTTATACGTCAA
		248	CTCGGGTCGGTGGCT | cTGGTAACGACCAGT
pB453	3360	3606	GGTTGCCCGGTGGCT | tTGGTTATACGTCAA
		247	GCTCGGGTCGGTGGC | TcTGGTAACGACCAG
pB253	3835	4081	CGGTACGGTCGGGGC | gCTGGTGTCGCCCGG
		247	GCTCGGGTCGGtGGC | tCTGGTAACGACCAG
pB554	3836	4072	CATGGTGGCCGGTAC | GGTCGGGGCgCTGGT
		237	AGAAGGGTTTGCTCG | GGTCGGtGGCtCTGG
pB353 / pB153 / pS221	4003	4401	TGATGGTGCTgGACA | CGCTGCGCCGGTTCC
		399	TGCCGTTGCTaGACA | TTGCCAGCCAGTGCC
pB653 / pB154 / pS321	5077	5361	ATCGCGCAGGCCGTC | aTGGGTGGCGGCCAG
		285	TCCCGGCTGGCCGTC | cTGGCCGCCACATGA
**Deletion plasmids (Del)**			
pBD3	7534	6912	TAGGATACAGAAACA | GAGGAGATATTACGG
		8776	TTAACAAGGGTATCA | CCCTCGAACTTCACT
pBD4	7938	7035	TCCCGCGTGGTGAAC | CAGGCCAGCCACGTT
		8495	GCTTTTCGTTGGGAT | CTTTCGAAAGGGCAG
pBD5	7267	6942	TACGCAAGTACACAA | GATACAGGAGAGGTA
		9073	ACATCACCGTCTAAT | TCCACGAGGATTGGG
pBD6	7744	7024	ATCAGACCGTTTCCC | GCGTGGTGAACCAGG
		8678	CCATAATGTACACAT | TATGGGAGTTATAGT

Evolved satellite plasmids are named as follows: with a letter for the experiment in which they were observed (pB, *in vitro E. coli*; pS, *in vitro S. alvi*; pC, *in vivo S. alvi*); then the index of the experimental population in which they were observed (1–10), then a digit for the transfer or time point at which they were isolated, and with a final digit representing a distinct clonal isolate from that population or sequenced PCR amplicon from that sample. Evolved deletion plasmids were all from the *E. coli* experiment and are labeled pBD with single unique index. Endpoint coordinates are for the terminal bases remaining in the pQGS sequence (Genbank:MH423581) that flank the deleted portion of each plasmid. The flanking sequences have a vertical bar showing the location where the deletion begins in the upper row and ends in the lower row. The portions of these flanking sequences that are shaded grey are deleted in evolved plasmids. Therefore, the sequence of the new plasmid maintains the bases on the left side of the upper row and then continues into the bases on the right side of the lower row. Microhomologies that appear to mediate the formation of satellite plasmids are highlighted in red with lowercase letters for mismatched bases.
