# Supplementary material for: Evolution of satellite plasmids can prolong the maintenance of newly acquired accessory genes in bacteria
Source: Nat Commun. 2019 Dec 20;10:5809. doi: 10.1038/s41467-019-13709-x (PMC6925257; doi:10.1038/s41467-019-13709-x)
Supplement: Supplementary file 3 — Reporting Summary [file 41467_2019_13709_MOESM3_ESM.pdf]

nature research

Corresponding author(s): Jeffrey Barrick

Last updated by author(s): Nov 15, 2019

Reporting Summary

Nature Research wishes to improve the reproducibility of the work that we publish. This form provides structure for consistency and transparency in reporting. For further information on Nature Research policies, see Authors & Referees and the Editorial Policy Checklist .

Statistics

For all statistical analyses, confirm that the following items are present in the figure legend, table legend, main text, or Methods section.

n/a	Confirmed	
☐	☒	The exact sample size (n) for each experimental group/condition, given as a discrete number and unit of measurement
☐	☒	A statement on whether measurements were taken from distinct samples or whether the same sample was measured repeatedly
☐	☒	The statistical test(s) used AND whether they are one- or two-sided<br>*Only common tests should be described solely by name; describe more complex techniques in the Methods section.*
☐	☒	A description of all covariates tested
☐	☒	A description of any assumptions or corrections, such as tests of normality and adjustment for multiple comparisons
☐	☒	A full description of the statistical parameters including central tendency (e.g. means) or other basic estimates (e.g. regression coefficient) AND variation (e.g. standard deviation) or associated estimates of uncertainty (e.g. confidence intervals)
☐	☒	For null hypothesis testing, the test statistic (e.g. F, t, r) with confidence intervals, effect sizes, degrees of freedom and P value noted<br>*Give P values as exact values whenever suitable.*
☐	☒	For Bayesian analysis, information on the choice of priors and Markov chain Monte Carlo settings
☐	☒	For hierarchical and complex designs, identification of the appropriate level for tests and full reporting of outcomes
☐	☒	Estimates of effect sizes (e.g. Cohen's d, Pearson's r), indicating how they were calculated

Our web collection on statistics for biologists contains articles on many of the points above.

Software and code

Policy information about availability of computer code

Data collection	R (statistics), breseq (Illumina genome sequenceing data analysis)
Data analysis	Python and R scripts for the repeat analysis and population genetics simulations are freely available online (https://github.com/barricklab/satellite-plasmid).

For manuscripts utilizing custom algorithms or software that are central to the research but not yet described in published literature, software must be made available to editors/reviewers. We strongly encourage code deposition in a community repository (e.g. GitHub). See the Nature Research guidelines for submitting code & software for further information.

Data

Policy information about availability of data

All manuscripts must include a data availability statement. This statement should provide the following information, where applicable:

- Accession codes, unique identifiers, or web links for publicly available datasets
- A list of figures that have associated raw data
- A description of any restrictions on data availability

The source data underlying Figs 1B–E, 2A–H, 3A and C, 4B–C, and Supplementary Figs 1B–C, 2, 4A–B, and 5 are provided as a Source Data file. Genome sequencing data are available from the NCBI Sequence Read Archive (SRP149032[https://www.ncbi.nlm.nih.gov/sra/?term=SRP149032]).

Field-specific reporting

Please select the one below that is the best fit for your research. If you are not sure, read the appropriate sections before making your selection.

Life sciences study design

All studies must disclose on these points even when the disclosure is negative.

Sample size	Sample sizes were chosen as large as experimentally feasible for evolution experiments, fitness competition assays, qPCR, PCR analysis of satellite plasmids, and whole-genome sequencing.
Data exclusions	No data were excluded
Replication	All samples used biological replication and qPCR additionally used technical replication for each biological replicate. We performed two replicates of the bee experiments (with five cages each time) to account for the larger expected variation in using this animal host.
Randomization	Our experiments had a simple design that did not require randomization.
Blinding	Blinding was not relevant

Reporting for specific materials, systems and methods

We require information from authors about some types of materials, experimental systems and methods used in many studies. Here, indicate whether each material, system or method listed is relevant to your study. If you are not sure if a list item applies to your research, read the appropriate section before selecting a response.

Materials & experimental systems

n/a	Involved in the study	
☒	☐	Antibodies
☒	☐	Eukaryotic cell lines
☒	☐	Palaeontology
☐	☒	Animals and other organisms
☒	☐	Human research participants
☒	☐	Clinical data

Methods

n/a	Involved in the study	
☒	☐	ChIP-seq
☐	☒	Flow cytometry
☒	☐	MRI-based neuroimaging

Animals and other organisms

Policy information about studies involving animals; ARRIVE guidelines recommended for reporting animal research

Laboratory animals	Honey bees were collected from hives kept at UT Austin
Wild animals	Not applicable
Field-collected samples	Not applicable
Ethics oversight	Not applicable

Note that full information on the approval of the study protocol must also be provided in the manuscript.

Flow Cytometry

Plots

Confirm that:

☒ The axis labels state the marker and fluorochrome used (e.g. CD4-FITC).

☒ The axis scales are clearly visible. Include numbers along axes only for bottom left plot of group (a 'group' is an analysis of identical markers).

☒ All plots are contour plots with outliers or pseudocolor plots.

☒ A numerical value for number of cells or percentage (with statistics) is provided.

Methodology

Sample preparation	Cells were washed and then counterstained with FM 4-64
Instrument	BD LSRFortessa
Software	Data were visualized with FlowJo but analyzed with R

Cell population abundance	Not applicable
Gating strategy	Only events with FM 4-64 signal were analyzed for GFP fluorescence

☐ Tick this box to confirm that a figure exemplifying the gating strategy is provided in the Supplementary Information.
