# Supplementary material for: Evolution of satellite plasmids can prolong the maintenance of newly acquired accessory genes in bacteria
Source: Nat Commun. 2019 Dec 20;10:5809. doi: 10.1038/s41467-019-13709-x (PMC6925257; doi:10.1038/s41467-019-13709-x)
Supplement: Supplementary file 4 — Description of Additional Supplementary Files [file 41467_2019_13709_MOESM4_ESM.pdf]

Description of Additional Supplementary Files

File Name: Supplementary Data 1
Description: Flow cytometry data
